# Supplementary material for: Impact of correcting misinformation on social disruption
Source: PLoS One. 2022 Apr 4;17(4):e0265734. doi: 10.1371/journal.pone.0265734 (PMC8979435; doi:10.1371/journal.pone.0265734)
Supplement: Attachment — Submitted filename: Response to Reviewers.rtf [file pone.0265734.s002.rtf]

013fDear Editor and Reviewers:
 Thank you for reviewing our paper, “Impact of correcting misinformation on social disruption.” We appreciate all the comments, which were constructive and helpful for improving the manuscript. Please refer to our responses below and the revised parts in the main paper. The revised parts are highlighted.
We trust that our revisions are satisfactory, but we are happy to consider further amendments if requested.


Reviewer #1
(1) The authors stated that, the major contribution of the study is the "impact of corrective information on society and clarifies its appropriate amount as against the previous studies that concentrated on the impact of corrective information on attitudes toward misinformation". I think the author need to give more clarification on the bases of their justification. For example, what are the issues in the previous studies that the authors were able to identify and improve upon in this study?
Response 1:
Thank you for your insightful comments. 
Our contributions can be briefly summarized as follows
-	1) We observed that people behaved as if they had believed misinformation even if the original misinformation was not spread to them. 
-	2) Our results suggest that such behavior is caused by pluralistic ignorance due to spreading excessive corrective information.
-	3) Under such a situation, we  used simulation experiments to show that the appropriate amount of corrective information to spread depends on the amount of misinformation spread.
We described the differences between previous studies and our study in more detail in the Discussion section (p.15-16, ll.554-596), in order to clarify our contribution. 

(2) The authors fails to make comparism of their methods with the existing models in the research domain to demonstrate the effectiveness of their methods.
Response 2:
The main contribution of our study is not the proposal of any new methods, but the detection of new phenomena. The method used is principal component regression, which is often used and works well. Therefore, it is not necessary for us to newly verify the effectiveness of this method.


Reviewer #2: 
# This paper need a lot of English editing and improving the flow of the work.
Response 1:
Thank you for providing important comments.
As for the English, the text was revised by a professional editing service.
Regarding the flow of the work, we have revised the text to match the typical section structure as follows. 
A description of the overall flow of this research has been added to the Materials and Methods section. We wrote about the statistical analysis in Study 1 and about the simulation in Study 2. In Study 1, we described the modeling method and its results. Study 2 covers the purpose, methods, results, and discussion of each of the three simulations.

# The Discussion section need to be expanded and more informative. The discussion of the main findings must be clear.
Response 2:
Our main findings are as follows:
-	1) We observed that people behaved as if they had believed misinformation even if the original misinformation was not spread to them. 
-	2) Our results suggest that such behavior is caused by pluralistic ignorance due to spreading excessive corrective information.
-	3) Under such a situation, we  used simulation experiments to show that the appropriate amount of corrective information to spread depends on the amount of misinformation spread.
We clarify these findings by discussing the consistencies and differences between our results and previous works (see the Discussion section (p.15-16,  ll.554-596)).

# The whole paper must be re-arranged and organized like this for example (Abstract, Introduction, Method, Results, Discussions and finally Conclusions. In the introduction the related works, the research gaps and the main objective on this work must be clearly reported. All things related to the dataset and the regression methods should be written in the methodology.
# I advised the authors to check some published examples in order to know how to arrange their work.

Response 3:
According to this comment, we revised the section structure of our manuscript based on the conventions in the literature (see Response 1).


Reviewer #3: 
1. Please reduce the Figure size and keep them clear.
Response 1:
Thank you for your helpful comments.
The size of the figures may be due to the submission system of PLOS ONE. When published, they will be of the appropriate size.

2. Please check few typing mistakes such as page no. 2 line no. 61.
Response 2:
We checked this and made the necessary revisions.

3. Please try to keep the tables near to the text so that it would be easy to read.
Response 3:
We checked and revised them.


Editor
1. Please ensure that your manuscript meets PLOS ONE's style requirements, including those for file naming. The PLOS ONE style templates can be found at 
https://journals.plos.org/plosone/s/file?id=wjVg/PLOSOne_formatting_sample_main_body.pdf and 
https://journals.plos.org/plosone/s/file?id=ba62/PLOSOne_formatting_sample_title_authors_affiliations.pdf
Response 1:
We checked and revised them.

2. We note that you have stated that you will provide repository information for your data at acceptance. Should your manuscript be accepted for publication, we will hold it until you provide the relevant accession numbers or DOIs necessary to access your data. If you wish to make changes to your Data Availability statement, please describe these changes in your cover letter and we will update your Data Availability statement to reflect the information you provide.
3. Thanks for stating that you'll provide the Tweet IDs upon accept.
Response 2:
We attached the IDs of tweets and retweets as supporting information.
Normal;heading 1;heading 2;heading 3;heading 4;heading 5;heading 6;heading 7;heading 8;heading 9;caption;Title;Subtitle;Strong;Emphasis;Placeholder Text;No Spacing;Light Shading;Light List;Light Grid;Medium Shading 1;Medium Shading 2;Medium List 1;Medium List 2;Medium Grid 1;Medium Grid 2;Medium Grid 3;Dark List;Colorful Shading;Colorful List;Colorful Grid;Light Shading Accent 1;Light List Accent 1;Light Grid Accent 1;Medium Shading 1 Accent 1;Medium Shading 2 Accent 1;Medium List 1 Accent 1;Revision;List Paragraph;Quote;Intense Quote;Medium List 2 Accent 1;Medium Grid 1 Accent 1;Medium Grid 2 Accent 1;Medium Grid 3 Accent 1;Dark List Accent 1;Colorful Shading Accent 1;Colorful List Accent 1;Colorful Grid Accent 1;Light Shading Accent 2;Light List Accent 2;Light Grid Accent 2;Medium Shading 1 Accent 2;Medium Shading 2 Accent 2;Medium List 1 Accent 2;Medium List 2 Accent 2;Medium Grid 1 Accent 2;Medium Grid 2 Accent 2;Medium Grid 3 Accent 2;Dark List Accent 2;Colorful Shading Accent 2;Colorful List Accent 2;Colorful Grid Accent 2;Light Shading Accent 3;Light List Accent 3;Light Grid Accent 3;Medium Shading 1 Accent 3;Medium Shading 2 Accent 3;Medium List 1 Accent 3;Medium List 2 Accent 3;Medium Grid 1 Accent 3;Medium Grid 2 Accent 3;Medium Grid 3 Accent 3;Dark List Accent 3;Colorful Shading Accent 3;Colorful List Accent 3;Colorful Grid Accent 3;Light Shading Accent 4;Light List Accent 4;Light Grid Accent 4;Medium Shading 1 Accent 4;Medium Shading 2 Accent 4;Medium List 1 Accent 4;Medium List 2 Accent 4;Medium Grid 1 Accent 4;Medium Grid 2 Accent 4;Medium Grid 3 Accent 4;Dark List Accent 4;Colorful Shading Accent 4;Colorful List Accent 4;Colorful Grid Accent 4;Light Shading Accent 5;Light List Accent 5;Light Grid Accent 5;Medium Shading 1 Accent 5;Medium Shading 2 Accent 5;Medium List 1 Accent 5;Medium List 2 Accent 5;Medium Grid 1 Accent 5;Medium Grid 2 Accent 5;Medium Grid 3 Accent 5;Dark List Accent 5;Colorful Shading Accent 5;Colorful List Accent 5;Colorful Grid Accent 5;Light Shading Accent 6;Light List Accent 6;Light Grid Accent 6;Medium Shading 1 Accent 6;Medium Shading 2 Accent 6;Medium List 1 Accent 6;Medium List 2 Accent 6;Medium Grid 1 Accent 6;Medium Grid 2 Accent 6;Medium Grid 3 Accent 6;Dark List Accent 6;Colorful Shading Accent 6;Colorful List Accent 6;Colorful Grid Accent 6;Subtle Emphasis;Intense Emphasis;Subtle Reference;Intense Reference;Book Title;Bibliography;TOC Heading;
